# STATegra: Multi-Omics Data Integration – A Conceptual Scheme With a Bioinformatics Pipeline

**DOI:** 10.3389/fgene.2021.620453

**Published:** 2021-03-04

**Authors:** Nuria Planell, Vincenzo Lagani, Patricia Sebastian-Leon, Frans van der Kloet, Ewoud Ewing, Nestoras Karathanasis, Arantxa Urdangarin, Imanol Arozarena, Maja Jagodic, Ioannis Tsamardinos, Sonia Tarazona, Ana Conesa, Jesper Tegner, David Gomez-Cabrero

**Affiliations:** ^1^Translational Bioinformatics Unit, Navarrabiomed, Complejo Hospitalario de Navarra (CHN), Universidad Pública de Navarra (UPNA), IdiSNA, Pamplona, Spain; ^2^Institute of Chemical Biology, Ilia State University, Tbilisi, Georgia; ^3^Gnosis Data Analysis P.C., Heraklion, Greece; ^4^Department of Genomic and Systems Reproductive Medicine, IVI-RMA (Instituto Valenciano de Infertilidad – Reproductive Medicine Associates) IVI Foundation, Valencia, Spain; ^5^Swammerdam Institute for Life Sciences, University of Amsterdam, Amsterdam, Netherlands; ^6^Department of Clinical Neuroscience, Karolinska Institutet, Center for Molecular Medicine, Karolinska University Hospital, Stockholm, Sweden; ^7^Institute of Computer Science, Foundation for Research and Technology-Hellas, Heraklion, Greece; ^8^Computational Medicine Center, Thomas Jefferson University, Philadelphia, PA, United States; ^9^Cancer Signalling Unit, Navarrabiomed, Complejo Hospitalario de Navarra (CHN), Universidad Pública de Navarra (UPNA), Health Research Institute of Navarre (IdiSNA), Pamplona, Spain; ^10^Computer Science Department, University of Crete, Heraklion, Greece; ^11^Department of Applied Statistics, Operations Research and Quality, Universitat Politècnica de València, València, Spain; ^12^Microbiology and Cell Science, Institute for Food and Agricultural Sciences, University of Florida, Gainesville, FL, United States; ^13^Genetics Institute, University of Florida, Gainesville, FL, United States; ^14^Biological and Environmental Sciences and Engineering Division, King Abdullah University of Science and Technology (KAUST), Thuwal, Saudi Arabia; ^15^Unit of Computational Medicine, Department of Medicine, Center for Molecular Medicine, Karolinska Institutet, Karolinska University Hospital, Stockholm, Sweden; ^16^Science for Life Laboratory, Solna, Sweden; ^17^Mucosal & Salivary Biology DivisionKing’s College London Dental Institute, London, United Kingdom

**Keywords:** multi-omic analyses, data-integration, next-generation sequencing, component analysis, non-parametric combination, GeneSetCluster

## Abstract

Technologies for profiling samples using different omics platforms have been at the forefront since the human genome project. Large-scale multi-omics data hold the promise of deciphering different regulatory layers. Yet, while there is a myriad of bioinformatics tools, each multi-omics analysis appears to start from scratch with an arbitrary decision over which tools to use and how to combine them. Therefore, it is an unmet need to conceptualize how to integrate such data and implement and validate pipelines in different cases. We have designed a conceptual framework (STATegra), aiming it to be as generic as possible for multi-omics analysis, combining available multi-omic anlaysis tools (machine learning component analysis, non-parametric data combination, and a multi-omics exploratory analysis) in a step-wise manner. While in several studies, we have previously combined those integrative tools, here, we provide a systematic description of the STATegra framework and its validation using two The Cancer Genome Atlas (TCGA) case studies. For both, the Glioblastoma and the Skin Cutaneous Melanoma (SKCM) cases, we demonstrate an enhanced capacity of the framework (and beyond the individual tools) to identify features and pathways compared to single-omics analysis. Such an integrative multi-omics analysis framework for identifying features and components facilitates the discovery of new biology. Finally, we provide several options for applying the STATegra framework when parametric assumptions are fulfilled and for the case when not all the samples are profiled for all omics. The STATegra framework is built using several tools, which are being integrated step-by-step as OpenSource in the STATeg**R**a Bioconductor package.^1^

## Introduction

Computational and experimental developments have enabled the profiling of multiple layers of cell regulation: genome, transcriptome, epigenome, chromatin conformation or metabolome, among many globally known “omics” (Ramos et al., 2017; Gomez-Cabrero et al., 2019). The development of such technologies was driven by the understanding that a single-omic does not provide enough information to allow dissecting biological mechanisms (Joyce and Palsson, 2006; Gomez-Cabrero et al., 2014). For instance, while specific DNA variations have been linked with multiple diseases, the associated mechanisms are not fully understood (Gilad et al., 2008; James et al., 2018). As a result, multi-omics data-sets are increasingly applied across biological domains such as cancer biology (Gerstung et al., 2015; Tomczak et al., 2015; Iorio et al., 2016; Mertins et al., 2016; de Anda-Jáuregui and Hernández-Lemus, 2020). Furthermore, single-cell multi-omics analysis (Macaulay et al., 2017; Colomé-Tatché and Theis, 2018; Chen et al., 2019; Welch et al., 2019) has just become a reality.

However, from the necessity of multi-omics profiling came the need for multi-omics analysis tools. Thus, integrative approaches are expected to generate significantly more comprehensive insights into the biological systems under study (SuS). A myriad of such tools in the literature may be categorized and classified differently (possibly in complex ways; Gomez-Cabrero et al., 2014; Hofmann-Apitius et al., 2015; Kannan et al., 2016; Meng et al., 2016; Rohart et al., 2017; Argelaguet et al., 2018; Stein-O’Brien et al., 2018). While each of the tools is a valuable resource for any multi-omics research, combining them into a *conceptually unified framework* is key. Equally important is the fact that each framework must be as generic as possible. Thus, we introduce the STATegra framework, in which we integrate three multi-omics based approaches *into a single pipeline*: (a) Component Analysis (CA) to understand the coordination among omics data-types (Måge et al., 2019); (b) Non-Parametric Combination (NPC) analysis to leverage on paired designs to increase statistical power (Karathanasis et al., 2016); and (c) an integrative exploratory analysis (Ewing et al., 2020). Furthermore, this framework may be extended by including additional tools such as network analysis (Barabási et al., 2011; Yugi et al., 2016). We incorporated most of these tools into the STATeg**R**a Bioconductor package to facilitate their use.^2^ The package is continuously being updated and developed. Furthermore, as described in the framework, additional tools are planned to be incorporated into the Bioconductor package, e.g., the pESCA (Song et al., 2020) for multi-omics CA and the GeneSetCluster (Ewing et al., 2020) for multi-omics exploratory analysis.

To demonstrate the added value of the STATegra framework as a whole, we applied it to two data-sets from The Cancer Genome Atlas (TCGA): the glioblastoma data-set (Turcan et al., 2012) and the melanoma data-set (Akbani et al., 2015). We also explored (i) the use of samples for which only a subset of omics profiles is available and (ii) the use of parametric vs. non-parametric analysis.

## Materials and Methods

Additional information is included in Supplementary Material, and an html-R Markdown document is provided for each data-set in Supplementary Material; each document provides a comprehensive overview of the code used to enhance their reproducibility.

### Downloading and Preprocessing Data

We selected the Glioblastoma Multiforme (GBM) and the SKCM data-sets from TCGA. The level 3 publicly available data for gene expression (gene expression calls), miRNA (miRNA expression calls), and DNA methylation (beta values per CpG, DNAm) were obtained per sample through the NCI’s Genomic Data Commons (GDC) portal (Tomczak et al., 2015). The associated metadata for each project was also obtained. Additionally, for the SKCM data-set, curated metadata generated in a previous TCGA study was also used (Akbani et al., 2015).

*Glioblastoma multiforme*: three data types were downloaded: array-based expression (mRNA) – Affymetrix Human Genome HT U133A, array-based expression (miRNA) – Agilent Microarray, and array-based DNA Methylation (DNAm) – Illumina Human Methylation 450 K. The number of available samples differed depending on the omic: mRNA, miRNA, and DNAm profiles are available for 523, 518, and 95 samples, respectively (Supplementary Table 1; Supplementary Figure 2A).

*Skin Cutaneous Melanoma*: three data types were downloaded: RNA-seq-based expression (mRNA) – Illumina HiSeq 2000, miRNA-Seq-based expression (miRNA) – Illumina HiSeq 2000, and array-based DNA Methylation (DNAm) – Illumina Human Methylation 450 K. The data from these three omics are available for all the individuals (*n* = 425); however, divergences between the initial date of diagnosis (driving the metadata information) and the TCGA specimen date were identified (Supplementary Figure 1). Consequently, we decided to include only those cases for which specimens were obtained within a 1-year window from diagnosis (*n* = 104).

Supplementary Table 1 describes the characteristics of the two data-sets and the pre-processing steps applied before starting the integrative workflow of multi-omics data. We conducted an exhaustive exploration for each data type assessing the need for data normalization and/or filtering (Supplementary Material). Metadata is available for GBM and SKCM (summarized in Supplementary Table 2 and described in Supplementary Tables 3, 4 for a detailed description of the variables). In general, the data provided by TCGA contains information on demographic features (age, gender, race, and ethnicity), tumor characteristics (age at diagnosis, the primary site of the disease, stage of the neoplasm, prior glioma, ulceration in melanoma, Karnofsky score for GBM, and Breslow thickness for SKCM), survival outcome (vital status, days to death, days to the last follow-up), and technical processes (batch number, tissue source site – TSS, i.e., centers which collect samples and clinical metadata).

At the end of the preprocessing, numerous matrices, i.e., one matrix per every omics data-type (mRNA, miRNA, and DNAm), plus one additional matrix containing the metadata of the samples, compose each data-set (GBM, SKCM). Omics data-type matrices are arranged placing measurements (a.k.a. features) on rows and samples in columns, while metadata matrices include samples as rows and metadata information (e.g., age, gender, etc.) in columns.

### Component Analysis for Two Data-Types (omicsPCA)

To perform joint exploration of data, the two data-types must fulfill the following criteria: (i) each feature must be scaled and (ii) only samples that are common to the two data types can be analyzed. Each feature was mean-centered and then normalized to the unit sum of squares (Frobenius normalization). Due to sample availability, component analysis for two data-type matrices was restricted for each analysis for common samples (Supplementary Figure 2A).

Once input data were ready, the two main omicsPCA steps were applied: model selection and subspace recovery. For model selection, we aimed to identify the correct model, which means the exact number of common (shared) components and the number of distinctive components per data-type. We investigated the following methodologies: JIVE (Lock et al., 2013; the jive R package), PCA-GCA (Gu and Van Deun, 2019; RegularizedSCA R package), and pESCA (Song et al., 2020; RpESCA and Rspectra R packages; Supplementary Table 5 and Supplementary html-R Markdown document).

Finally, the association between metadata and the shared/individual components obtained was assessed using the Kruskal-Wallis test, Spearman’s correlation, or the Cox regression model, depending if the variable of interest was categorical, numerical or time-to-event, respectively.

All analyses were conducted in R (R Core Team, 2017).

### Non-Parametric Combination for Two Data-Types (omicsNPC)

Non-Parametric Combination techniques allow combining statistical evidence (*p*-values) across data-types to obtain a more precise characterization of the changes associated with the outcome of interest (Karathanasis et al., 2016).

The above-described approach allows to integrating data matrices defined on overlapping sets of samples. Taking advantages of this possibility, we explored the NPC following two strategies: analyzing only common samples or analyzing all available samples (including non-overlapping ones, when applicable).

Importantly, NPC methods require linking the features across data-types. To that end, the relation between mRNA and miRNA and mRNA and methylation were obtained using the *SpidermiR* R package (Cava et al., 2017) and RGmatch (Furió-Tarí et al., 2016), respectively.

In the case of mRNA and miRNA mapping, different versions of annotation were found; we combined the following two: the *miRNAmeConverter* (Haunsberger et al., 2017) and *anamiR* (Wang et al., 2019) R packages.

Finally, the NPC may be run using the *omicsNPC* function from the STATeg**R**a package using the two data-types, the mapping file (i.e., mRNA – miRNA), and the variables to include in the model (see R-code below) as inputs.

In our analyses, the outcomes of interest were survival for the GBM data-set and the primary site of tumor for the SKCM data-set. Additionally, age was included as a co-variable in all the models. Depending on the nature of the outcome of interest the analysis performed during NPC differs. In the case of GBM, the association between each molecular quantity and the time-to-event was assessed through a Cox Regression model (Cox, 1972). Since age is by itself a relevant factor (Supplementary Figure 5), it was treated as a time-varying factor by specifying a time-transform function (Therneau et al., 2020). In SKCM associations between each molecular quantity and the primary site of the tumor were assessed through a differential expression analysis using Limma (Robinson, 2009; highlighted lines from the R-code).

-------CODE-------

# Detailed version of the code is provided as SupplementaryMaterial (RMarkDown)

#NPC input

*mRNA_data* #mRNA expression data matrix

*miRNA_data* #miRNA expression data matrix

*mapping_gene* #mapping of mRNA to genes

*mapping_mirna* #mapping of miRNA to genes

#1 – Generate the mapping between mRNA and miRNA; a data frame describing how to map measurements across data-sets

*dataMappingExprMirna <- combiningMappings (mappings = list(expr = mapping_gene, mirna = mapping_mirna), retainAll = TRUE, reference = ‘Gene’)*

#2 – Specify data type.

# The type of analysis to be performed is defined here.

# For GBM, as the output of interest is the survival outcome, we must define a coxph function that considers the age as a co-variable. This defined function is called “ttCoxphContinuous”

*dataTypesExprMirna <- list(ttCoxphContinuous,ttCoxphContinuous)*

#For SKCM, as our output of interest is the differential expression between primary site of tumor, it is only necessary to define that our data-types are continuous.

*dataTypesExprMirna <- c(expr = ‘continuous’, mirna = ‘continuous’)*.

#3 - Preparing the data-sets as an ExpressionSet object (outcome variable refers to our variable of interest, in that case, “survival” for GBM data-set and “primary site of tumor” for SKCM data-set).

*mRNA <- createOmicsExpressionSet(Data = mRNA_data, pData = metadata[,c(“age”,“outcome”)]0029*

*miRNA <- createOmicsExpressionSet(Data = miRNA_data, pData = metadata[,c(“age”“outcome”)])*

*dataInputExprMirna <- list(expr = mRNA, mirna = miRNA)*

#4 - Setting methods to combine *p*-values

*combMethods <- c(“Fisher”,“Liptak”,“Tippett”)*

# Setting number of permutations

*numPerms <- 1000*

# Setting number of cores

*numCores <- 4*

# Setting omicsNPC to print out the steps that it performs.

*verbose <- TRUE*

#Run the omicsNPC

*omicsNPC_output <- omicsNPC(dataInput = dataInputExprMirna,*

*dataMapping = dataMappingExprMirna,*

*dataTypes = dataTypesExprMirna,*

*combMethods = combMethods,*

*numPerms = numPerms,*

*numCores = numCores,*

*verbose = verbose)*

-------------------------

### GeneSetClustering

Significant genes from omicsNPC in the different approaches (Adj.value of *p* < 0.05 or Fisher *p*-value <0.05 in NPC) were uploaded to the Ingenuity Pathway Analysis (IPA; Krämer et al., 2014) database (Qiagen), and core expression analysis was performed to identify affected canonical pathways and functional annotations. Right-tailed Fisher’s exact test was used to calculate a *p*-value. Canonical pathways/functional annotations were clustered together using *GeneSetCluster* (Ewing et al., 2020). Briefly, the gene-sets were grouped into clusters by calculating the similarity of pathways/annotations of the gene content using the relative risk (RR) of each e-set appearing with each other. Only significant gene-sets (values of *p* < 0.05) with a minimum of three genes were selected for functional exploration. RR scores were clustered into groups using k-means with the optimal number of genes determined using gap statistics.

## Results

**Figure 1:**
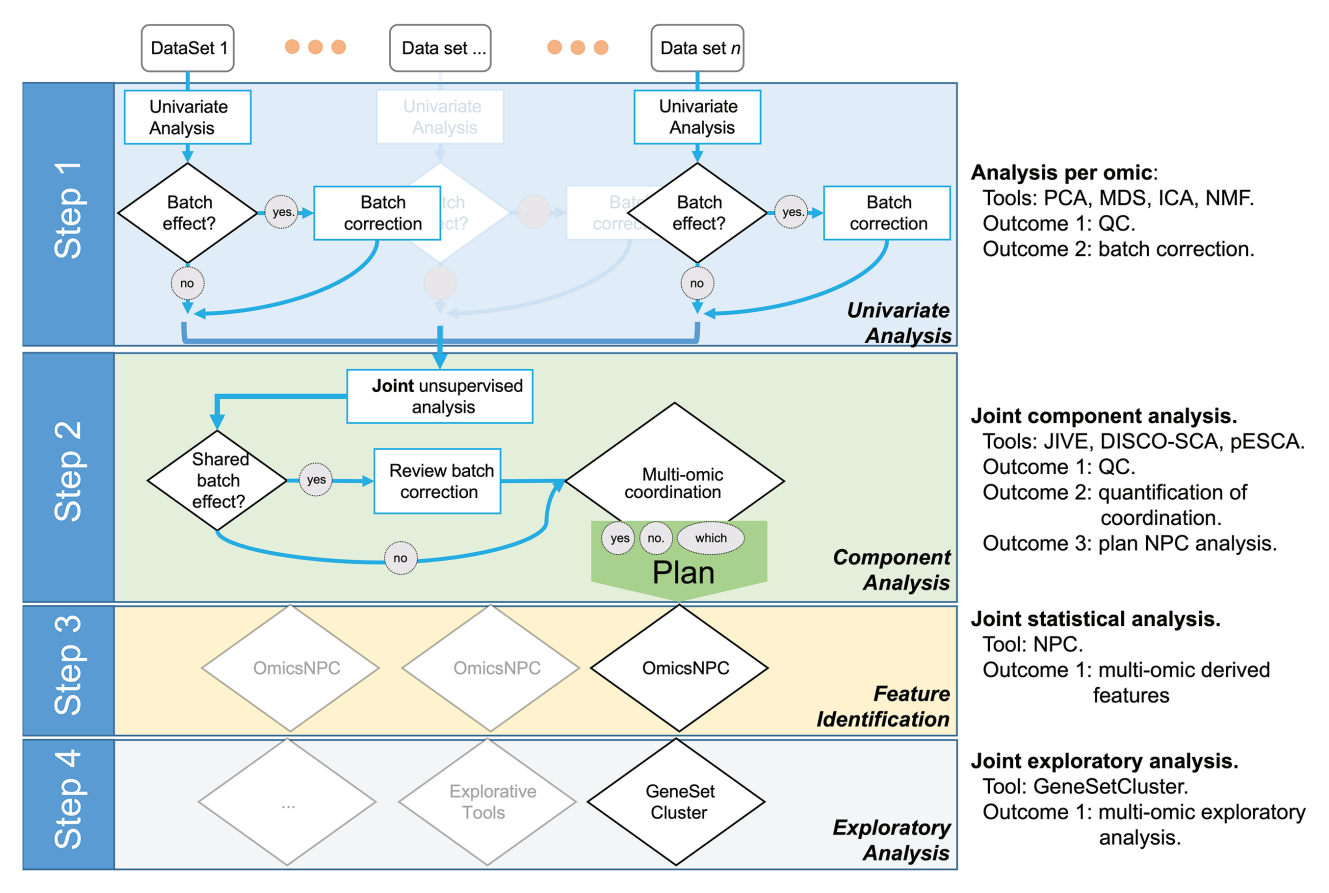
Workflow diagram of the multi-omics analysis framework.

We designed the STATegra framework as a four-step analysis (Figure 1). In the first step, each data-type was analyzed separately using state-of-the-art tools for each omic. Next, in a second step, we explored the shared variability between the different data-types using unsupervised techniques such as *Joint and Individual Variation Explained* (JIVE; Lock et al., 2013), implemented in OmicsPCA. This analysis provided qualitative and quantitative insights into how much the different data-types (e.g., different omics) and their features were “*coordinated*.” Moreover, the analysis provided useful information for targeting specific omics combinations (Gomez-Cabrero et al., 2019). In the third step, for those combinations of omics characterized as *coordinated*, NPC analysis allowed increasing the statistical power to identify significant features as we have recently demonstrated (Ewing et al., 2019; Fernandes et al., 2019). For that purpose, we used the NPC within the omicsNPC function (Karathanasis et al., 2016). In the final step, clustering tools (e.g., OmicsClustering) and gene-set enrichment analysis summarizing tools (such as GeneSetCluster, Ewing et al., 2020) allowed an integrated approach.

### Selected Case Studies

We selected two case studies: GBM and SKCM. GBM is the first cancer studied by TCGA (McLendon et al., 2008; Brennan et al., 2013). The TCGA GBM data-set consists of primary tumor samples from roughly 600 cases. The data-set contains gene expression, miRNA, and DNA methylation microarrays. Several findings have been reported on these data, including a molecular classification of glioblastoma based on gene expression profiles (classical, proneural, neural, and mesenchymal; Verhaak et al., 2010). The TCGA Consortium published the landscape of SKCM in 2015 (Akbani et al., 2015). The TCGA SKCM data-set consists of melanoma samples from patients diagnosed with either primary or metastatic cutaneous melanoma or metastatic melanoma of unknown primary from ~400 cases. The data-set contains genotype information, gene expression, and methylation microarrays. Based on these data, several findings have been reported, including the genomic identification of four mutant subtypes (BRAF hotspot, NF1 mutant, RAS hotspot, and triple wild-type) and a molecular classification based on gene expression profiles (immune, keratin, and MITF-low related profiles) associated with survival time. In general, patients from both studies were Caucasian with a median age of 58–59 years and a higher proportion of males (~60%). The mortality rate in GBM was high (78%) with a median life expectancy of around 1 year. For SKCM, 42% of patients died during follow-up and median life expectancy was of 1 year and 3 months (Supplementary Tables 1, 2).

### Step 1: Independent Data-Type Exploration and Characterization

Once the data is pre-processed, we recommend conducting quality controls for each individual data-type as the first step in the STATegra framework. In our example we made use of principal component analysis (PCA) as an unsupervised exploratory analysis. However, other matrix-factorization techniques may be used, e.g., Independent Component Analysis (ICA; Lee and Batzoglou, 2003) or Non-negative Matrix Factorization (NMF; Lee and Seung, 1999). It is important to emphasize the relevance of setting up a proper study design to avoid possible batch-effects not to be confounded with the biological effects under study: a component analysis will not overcome a wrong design.

In the GBM data-set case, the two first PCA components showed a limited amount of variability explained for all omics (Supplementary Figure 2B), suggesting a large per sample variability. As expected from the original TCGA publication (Verhaak et al., 2010), we found a significant association between the previously defined “gene expression subtypes” (Verhaak et al., 2010) and the first PCs of mRNA (Bonferroni adjusted value of *p* < 0.001; refer to Supplementary Material). Interestingly, such association was also found for miRNA and DNAm (Supplementary Figure 2C; adjusted value of *p* < 0.005). Moreover, we identified several clinical variables associated with at least one of the first three main components of omics data (refer to Supplementary Material; Bonferroni adjusted value of *p* < 0.05): survival outcome (mRNA, miRNA, DNAm) and TSS (mRNA).

In the case of the SKCM data-set, the two first PCA components showed a limited amount of variability explained for all omics (Supplementary Figure 3A). We identified several clinical variables associated with at least one of the first three main components of omics data (refer to Supplementary Material; Bonferroni adjusted value of *p* < 0.05): primary site of disease (mRNA, miRNA), neoplasm (mRNA), and pathological stage of the disease (mRNA, miRNA).

It is worth noting that some of the clinical variables were associated with at least one of the first three components in the individual data-type exploration *for more than one omics* data type. Such results apply to both GBM and SKCM data-sets. Consequently, we hypothesize that several omics are coordinated and their analytical integration would bring more statistical power and synergistic insights. In Step 2, we investigated such assumptions.

### Step 2: Joint Exploration and Characterization

As previously shown, several clinical variables were associated with more than one omics data-type in both selected data-sets. Such observations may indicate that some (if not all) those omics profiles are coordinated (or at least some of their features are). Therefore, the next step in the STATegra framework was to investigate and quantify a potential coordination.

**Figure 2:**
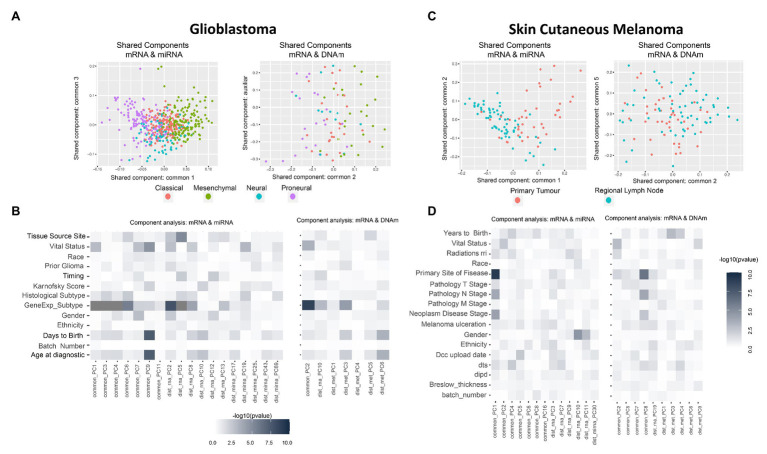
Multi-omics component analysis. **(A,C)** Component-based representation of Glioblastoma Multiforme (GBM) and Skin Cutaneous Melanoma (SKCM) joint exploration; mRNA + miRNA (left) and mRNA + methylation (right). First and second common components (or auxiliary if only one common component is found) are shown. Samples are colored based on gene expression subtype for GBM and primary site of disease for SKCM. **(B,D)** Heatmap representation of –log10 (*p*-values) of the statistical test between metadata and common and distinctive components of GBM and SKCM joint analysis; mRNA + miRNA (left) and mRNA + methylation (right). Color ranges from white to black, understood as *p*-values with no significance to significant *p*-values. Based on the nature of the variables, *p*-values were obtained by association, correlation, or using a survival test. *Radiations rri*, *dts*, and *dipd* denote, respectively, “*Radiations radiation regimen indication*,” “*Days to submitted specimen dx*,” and “*Date of initial pathologic diagnosis*.” See Supplementary Tables 3, 4 for a detailed description of the variables.

Thus, instead of looking at the PCA-derived components of mRNA and miRNA separately, we investigated the existence of components (or factors) shared by both omics (Gomez-Cabrero et al., 2019). Intuitively, while in PCA we projected using the main components per omic (refer to Supplementary Figures 2B, 3A as examples), we next aimed to identify projections where the components are informative for more than one data-type simultaneously (refer to Figures 2A,C). In summary, when analyzing the variability of data-types A and B, we aimed to identify components associated to both A and B (shared components), components associated only to A, and components associated only to B (distinctive components).

Multi-data-set component analysis methodologies have three key steps: (a) model selection, (b) subspace recovery, and (c) estimation of robustness. In (a) model selection, we aimed to identify the correct model, which means the exact number of common (*shared*) components and the number of *distinctive* components per data-type. The determination of model selection, although fundamental, remains an open question (van der Kloet et al., 2016; Måge et al., 2019); hence, no final function has yet been included in the STATeg**R**a package. However, we explored several methods [JIVE (Lock et al., 2013), PCA-GCA (Smilde et al., 2017), and pESCA (Song et al., 2020)]. Both, *common* and *distinctive* components obtained for each method are summarized in Supplementary Table 5. In our experience, the selected method depends on the nature of the data [as shown in (Måge et al., 2019)]. We do however recommend the use of several methodologies to establish more robust insights. While identifying the best model is an open challenge, we considered – based on the estimates – using the results from pESCA (Song et al., 2020), specifically pESCA (1%). Once the number of *shared* and *distinctive* components was determined, the subspace recovery (identification of loads and scores for the components) should be conducted using the same methodology used to identify space. Finally, to address robustness estimation we refer to the method in Måge et al. (2019).

In the current data-sets we were prioritizing a gene-centric analysis for both data-sets (GBM and SKCM); therefore, we posed two scenarios; the joint analysis of mRNA and miRNA, and the joint analysis of mRNA and methylation. We acknowledge that there are tools in development for integrating more than two omics; see for instance (Srivastava et al., 2013) and its application in Gomez-Cabrero et al. (2019).

*GBM data-set*: we identified seven shared components between mRNA and miRNA and one between mRNA and DNAm (refer to Supplementary Table 5). Figure 2A shows two PC score plots; the association between components (shared and distinctive) and clinical variables is shown in Figure 2B. After investigating all pairs of “share components vs. factors,” we observed that at least one shared component was significantly associated (Bonferroni adjusted value of *p* < 0.05) with: “gene expression subtype” derived from (Verhaak et al., 2010; mRNA-miRNA, mRNA-DNAm), survival outcome (mRNA-miRNA), and age (mRNA-miRNA; Figure 2B). No significant relationship was seen between gene expression subtype and survival outcome (Supplementary Figure 4, value of *p* = 0.06), although a relationship between age and survival outcome was observed (adjusted value of *p* <0.05). Based on these results, we hypothesized a coordination between the mRNA and miRNA profiles, and such coordination is associated with survival. Consequently, we also considered that integrating both data types will contribute to increasing the knowledge regarding GBM survival. We identified a limited global coordination when considering the mRNA and DNAm profiles.

*SKCM data-set*: seven shared components were identified between mRNA and miRNA profiles, and four common components between mRNA and DNAm profiles (refer to Supplementary Table 5). Figure 2C shows two PC score plots, and the association between components (shared and distinctive) and clinical variables is shown in Figure 2D. At least one component identified is significantly associated with the primary site of the disease for both mRNA-miRNA and mRNA-DNAm pairs and the disease stage for the mRNA-miRNA pair (refer to Supplementary Material; Bonferroni adjusted value of *p* <0.05). Based on these results, we concluded that mRNA, miRNA and DNAm are globally coordinated, and this is mainly associated with the primary site of the disease. Therefore, the integration of the three data-types may contribute to increase the knowledge on SKCM primary site.

Importantly, based on the *complexity of the data*, the joint exploration may allow data-type specific related batch effects (identified in Step 1) from batch effects associated with sample collection (which will be associated to all omics). Interestingly, more than two omics (*blocks*) can be analyzed to identify shared components (Srivastava et al., 2013; Argelaguet et al., 2018; Song et al., 2020).

The next challenge, Step 3, was to leverage the coordination identified among omics to gain statistical power to identify the relevant features that explain the SuS.

### Step 3: Integrative Differential Analysis, omicsNPC

In Step 3 we used NPC to increase the statistical power for the analysis of the SuS (Pesarin and Salmaso, 2010). Briefly, NPC non-parametrically combines *p*-values from associated features, such as a miRNA and one of its target genes measured on overlapping sets of samples. We used the omicsNPC (Karathanasis et al., 2016) included in the STATeg**R**a package, specifically tailored for the characteristics of omics data.

The main advantages of the NPC include: (a) high statistical power with minimal assumptions; (b) wide applicability on different study designs; (c) it allows integrating data modalities with different encodings, ranges, and data distributions; and (d) it models the correlation structures present in the data producing unbiased/calibrated *p*-values, an interpretable metric (Pesarin and Salmaso, 2010).

OmicsNPC first analyses each data-type separately through a permutation-based scheme. Currently, omicsNPC uses the package limma or survival (coxph) for computing statistics and *p*-values; however, the user may also customize the functions (refer to “Materials and Methods”). The resulting permuted-based *p*-values may be combined using Tippett’s (aimed to identify findings supported *by at least one omics modality*), Liptak’s (*by most omics modalities*), or Fisher’s (intermediate behavior between Tippett and Liptak) combination function. Following the original NPC, omicsNPC (Karathanasis et al., 2016) makes minimal assumptions: as permutation is employed throughout the process, no parametric form is assumed for the null distribution of the statistical tests, and the main requirement is that samples are freely exchangeable under the null-hypothesis. This frees the researcher from the need of defining and modeling between dataset dependencies. Most importantly, it provides global *p*-values for assessing the overall association of related features across different data modalities with the specified outcome (Pesarin and Salmaso, 2010).

*GBM analysis*: we aimed to investigate GBM survival through its relationship with omic features corrected for age, based on the association identified in Supplementary Figure 5. We only used samples profiled for all data-types (*n* = 515 and *n* = 83 for the mRNA-miRNA and mRNA-DNAm pairs, respectively). Table 1 (*Overlapping samples* column) presents the NPC outputs. When the NPC is applied on “mRNA and miRNA,” the integration allowed identifying 23 new genes and four new miRNAs. For “mRNA and DNAm,” the integration allowed identification of 106 new genes and 150 new CpG sites.

**Table 1:** Non-parametric combination analysis results of two-omics data from the GBM and SKCM projects.

	GBM		SKCM
mRNA + miRNA	Overlapping samples	Whole dataset	
mRNA dimension	7.814 × 515	7.814 × 523	9,491 × 104
mRNA significant	**1**	**4**	**216**
miRNA dimension	325 × 515	323 × 518	239 × 104
miRNA significant	**1**	**1**	**6**
mRNA-miRNA total pairs	24,665	24,665	20,225
NPC_Fisher significant pairs	27	50	114
New mRNA from NPC	**23**	**43**	**48**
New miRNA from NPC	**4**	**7**	**14**
mRNA + DNAm
mRNA dimension	9,620 × 83	9,620 × 523	9,564 × 104
mRNA significant	**2**	**7**	**277**
Methylation dimension	57,645 × 83	57,645 × 95	55,729 × 104
Methylation significant	**1**	**0**	**12**
mRNA-methylation total pairs	57,645	57,645	55,729
NPC_Fisher significant pairs	150	332	432
New mRNA from NPC	**106**	**174**	**116**
New methylation sites from NPC	**150**	**332**	**428**

Significance was considered for a False Discovery Rate <0.05. Bold values highlight the number of significant features.

*SKCM analysis*: we explored the omics characterization associated to the primary site of the disease. When the NPC was applied on “mRNA and miRNA,” the integration allowed identifying 48 new genes and 14 new miRNAs. For “mRNA and DNAm,” the integration allowed identifying 116 new genes and 428 new CpG sites. This increase of the statistical power was expected based on the results from the joint exploration (Figure 2).

### Alternatives to Step 3

*Including samples available for a sub-set of data-types*: when doing the NPC analysis, we considered samples available for both omics. However, in the case of GBM we discarded a large number of samples. In (Karathanasis et al., 2016; Ewing et al., 2019), we modified the NPC permutation protocol to include the discarded samples. We observed that the use of all samples allowed us to identify a larger number of novel features (“mRNA and miRNA” identified 43 new genes instead of 23; for complete results refer to Table 1, Column *Whole data-set*).

*Parametric version*: The NPC requires a large number of permutations, which is time consuming. To address this, the STATeRra package includes a parametric combination methodology (Benjamini and Heller, 2008; Karathanasis et al., 2016). This parametric approach is a faster alternative to NPC, which we suggest to use in preliminary explorations. In our analyses, the parametric approach generated a larger number of significant results in comparison to the non-parametric counterpart (Supplementary Table 6), which may be explained by unaccounted inter-data-sets correlations that inflate the significance of the p-values.

### Step 4: Exploratory Analysis and Determination of the Framework’s Added Value

**Figure 3:**
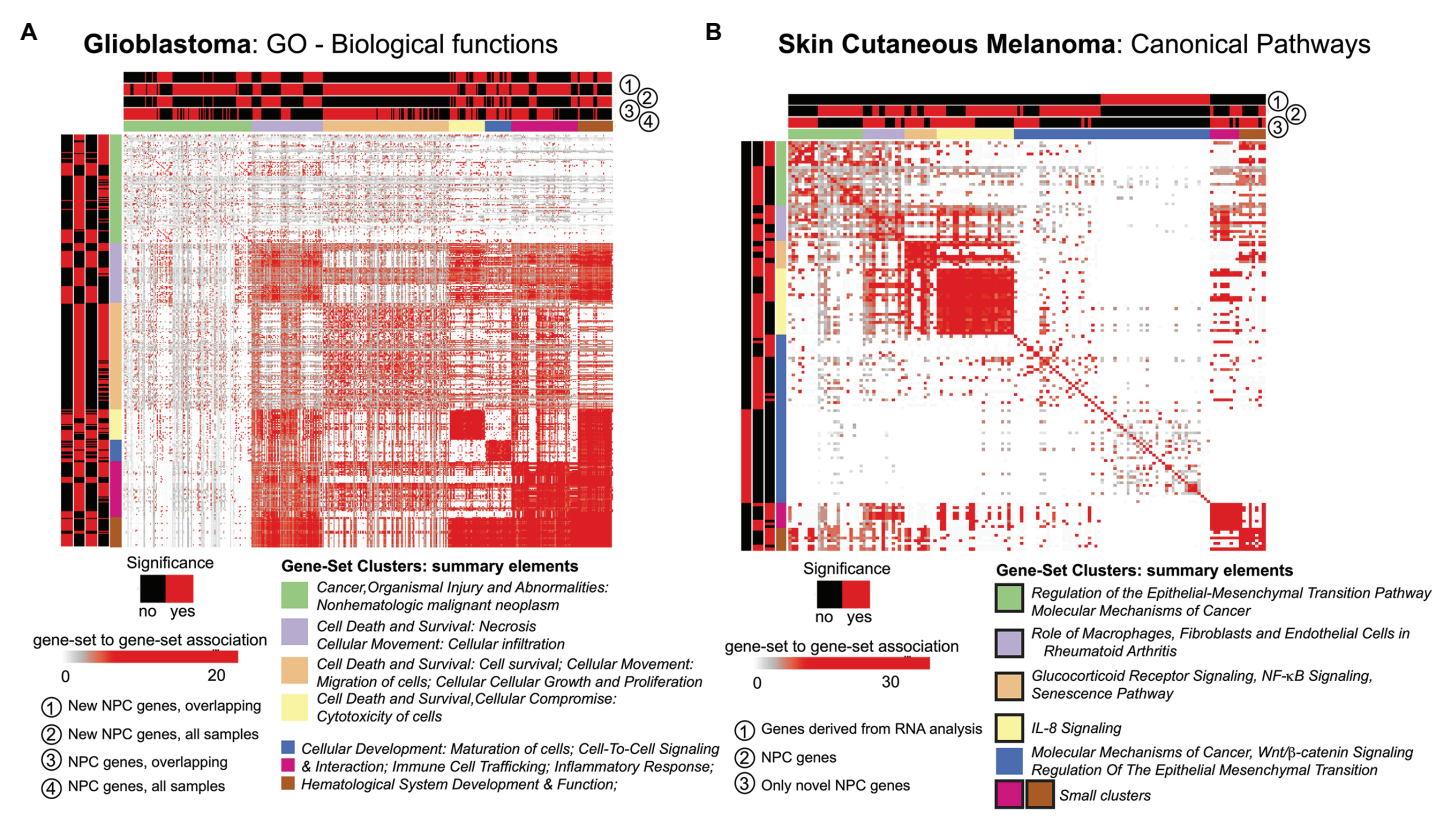
GeneSetCluster analysis. Each heatmap depicts the gene-set to gene-set RR distance (Ewing et al., 2020). In each case several gene-set analyses have been conducted. Red (yes)/black (no) shows which gene-sets have been identified for each gene-set analysis. **(A)** GBM and **(B)** SKCM. In SKCM, clusters presented as black lines are those associated to discoveries through multi-omics integration.

The STATegra framework provided novel genes, miRNAs, and CpG sites for the two selected cases in comparison to unimodal analyses. We investigated if such novel elements could also provide new insights at gene-set level. For this, we made use of the GeneSetCluster (Ewing et al., 2020), a tool that summarizes gene-set analysis (GSA) results derived from multiple analyses. It allows identifying core-results by clustering gene-sets and posterior exploration; furthermore, it analyzes the integration of more than one gene-set (which could be derived from more than one omic) simultaneously. When investigating SKCM, we compared three GSA: (Ramos et al., 2017) using genes derived from mRNA single-omic analysis, (Gomez-Cabrero et al., 2019) using genes derived from mRNA-miRNA NPC analysis, and (Gomez-Cabrero et al., 2014) genes in (Gomez-Cabrero et al., 2019) not identified in (Ramos et al., 2017). We observed that the set of genes in (Ramos et al., 2017) identified several relevant canonical pathways, which are also identified in (Gomez-Cabrero et al., 2019) and (Gomez-Cabrero et al., 2014); but, especially, (Gomez-Cabrero et al., 2014, 2019) GSA identified many additional relevant pathways as shown in Figure 3B for Canonical Pathways analysis (see box strokes on clusters). In the case of GBM, four GSAs were conducted with the following pair combinations: (a) “*considering only samples with all omics available* (OVERLAP)” or “*considering all samples* (ALL),” and (b) “*considering all identified genes*” or “*considering genes only identified by NPC*.” We observed major differences in the summarized gene-sets between OVERLAP vs. ALL; see for instance Figure 3A, when analyzing “Gene Ontology – Biological Functions” (Blake et al., 2015). The use of GeneSetCluster allowed us to demonstrate the added value of the STATegra framework. Furthermore, it is also a tool for multi-omics GSA integrative analysis that we consider as part of the STATegra framework. We plan to integrate such tools continuously to the STATeg**R**a package.

## Discussion

There are many bioinformatics integrative tools (Gomez-Cabrero et al., 2014; Yugi et al., 2016; Hasin et al., 2017; Argelaguet et al., 2018; Shafi et al., 2019). However, when carrying out multi-omics analysis, as a rule, researchers use custom pipelines that combine some of the available tools. While every multi-omics data combination is different, we believe that a general framework is key to gain knowledge for an “*optimized*” integrated research analysis in the future. We here present the STATegra framework, a multi-omics integrative pipeline, the result of integrative analyses done over the last decade (Karathanasis et al., 2016; Carlström et al., 2019; Ewing et al., 2019, 2020; Fernandes et al., 2019). In the two chosen case studies used to evaluate the STATegra framework, GBM and SKCM, we show through a consecutive four-step process (Figure 1), how single omics integration generates additional information. Step 2, Component Analysis, quantifies the coordination of the different data-types, a key phase to identify where omic-combination can be leveraged, and Step 3 -Non-Parametric Combination is used to gain statistical power. In both case studies, we detect a greater number of genes as shown in Table 1. Interestingly and following the gene expression vs. DNA Methylation relation, in the case of the statistically significant pair of features identified in the mRNA-DNAm analysis, were showing a bimodal – but mostly negative – distribution of the correlation between gene expression and DNA methylation (see Supplementary Figure 6). Step 4 examines the added value of the biological-insights of the features identified by the integration process.

In GBM we examine the association of the omics profiles with survival. In comparison to single-omic analysis, the STATegra framework identifies additional genes already known to be associated with GBM such as CAST, ATF5, GANAB [glycoprotein associated with GBM cancer stem cells (Dai et al., 2011)], ICAM [overexpressed in bevacizumab-resistant GBM (Piao et al., 2017)], CORO1A [upregulated in GBM (Berezovsky et al., 2014)], LYN [*in vitro* association of enhanced survival of GBM cells (Liu et al., 2013)], MET (proto-oncogene) and STAT5 [enhances GBM cells migration, survival (Roos et al., 2018), and proliferation (Feng and Cao, 2014)], among others. Most have been previously associated with cancer and particularly to glioblastoma. We also compare the identified miRNAs with existing miRNA-derived survival signatures (Srinivasan et al., 2011); only miR222 is identified in the single-omic analysis, while three additional miRNAs (miR31, miR221, and miR200b) are identified by STATegra.

With the analysis of GSA, STATegra identifies new gene-sets, e.g., the TREM1 signaling pathway, previously associated with GBM (Kluckova et al., 2020). In SKCM we investigated the omics association with the primary site of disease. In addition to the newly identified genes (refer to Table 1), the major STATegra-associated novel insights are derived from GSA analysis as shown in Figure 3B, particularly regarding the identification of the IL8 signaling, which is known to be relevant in SKCM (Shoshan et al., 2016; Tobin et al., 2019).

Importantly, the new results are not derived only because of the application of the tools, but also because the application of their combination as a framework (see also Figure 1). For instance, the outcome of the Component Analysis provides insights into which clinical variables to investigate or which combination of omics to prioritize in the next steps. Furthermore, as shown, the outcome of the NPC (identification of features by a single-omic or by paired-multi-omic-features) can be leveraged in the GeneSetCluster tool to identify pathways derived from single-omic or coordinated among omics as shown in Figure 3. Adding new tools to the framework or modifying the existing ones should aim to generate greater synergies between the selected tools.

It is important to point out that we are not comparing our analysis against the original publications: GBM (McLendon et al., 2008; Brennan et al., 2013) and SKCM (Akbani et al., 2015). The idea is to compare a generic framework with single-omic approaches. Moreover, since the questions and data-sets used are different from those in the original TCGA publications, a back-to-back comparison is not justified.

The results generated by STATegra show the *added value* of a general integrative framework. Still, we acknowledge that, similarly to Operations Research there is “*no-free-lunch*” (Wolpert and Macready, 1997). Generic frameworks provide an initial approximation to any integrative analysis. Once completed, they may be further customized – and therefore further optimized – to account for the characteristics of the data and considered SuS. Still, the STATegra framework’s value is its solid integration starting point, and - after being applied in many projects – generic rules can be extracted to allow an easier and faster customization.

Frameworks as the one we present here or complementary ones aimed to supervised learning (Rohart et al., 2017) are becoming increasingly necessary due to the amount of growing multi-omics data, particularly in the context of single-cell multi-omics (Colomé-Tatché and Theis, 2018). Further developments are required in multi-omics visualization (González et al., 2012), simulated data (Martínez-Mira et al., 2018), or further exploitation of Component Analysis as shown in (Stein-O’Brien et al., 2018), among others. Thus, we consider that the STATegra framework is the starting point that will be further developed over time. The next immediate steps are the inclusion of pESCA (Song et al., 2020) for multi-omic component analysis and GeneSetCluster (Ewing et al., 2020) for multi-omic exploratory analysis within the STATeg**R**a Bioconductor package.

## Data Availability Statement

Publicly available datasets were analyzed in this study. This data can be found at: TCGA Data Portal.

## Ethics Statement

The studies involving human participants were reviewed and approved by TCGA. The patients/participants provided their written informed consent to participate in this study.

## Author Contributions

DG-C, JT, AC, and ST designed the global pipeline. NP implemented the global pipeline and conducted the analysis. NP and DG-C wrote the manuscript. VL, PS-L, FK, EE, NK, and AU implemented specific parts of the analysis and provided supervision. All the authors reviewed the manuscript and were part of the review of the results of the analysis. All authors contributed to the article and approved the submitted version.

### Conflict of Interest

VL and IT were employed by Gnosis Data Analysis P.C., Greece.

The remaining authors declare that the research was conducted in the absence of any commercial or financial relationships that could be construed as a potential conflict of interest.

## Footnotes

^2^https://bioconductor.org/packages/release/bioc/html/STATegra.html
