# Supplementary material for: STATegra: Multi-Omics Data Integration – A Conceptual Scheme With a Bioinformatics Pipeline
Source: Front Genet. 2021 Mar 4;12:620453. doi: 10.3389/fgene.2021.620453 (PMC7970106; doi:10.3389/fgene.2021.620453)
Supplement: Supplementary file 2 [file Data_Sheet_2.pdf]

Supplementary Tables

STATegRa manuscript

Supplementary Table 1.

	Technology	Initial Data Dimension	Type	Filters	Transformation	Batch Correction	Final Data Dimension
**Glioblastoma Multiforme**							
**mRNA**	Affymetrix Human Genome HT U133A	12,042 x 548	Normalized log2-Expression signal	• Remove replicated samples<br>• Remove samples associated to a unique batch (1 batch – 1 samples)	No	Parametric ComBat	12,042 x 523
**miRNA**	Agilent Microarray	534 x 571	Normalized log2-expression signal	• Remove replicated samples<br>• Remove samples associated to a unique batch (1 batch – 1 samples)	No	Parametric ComBat	534 x 518
**Methylation**	Illumina Human Methylation 450K	485,577 x 154	Beta values	• Remove features with missing values<br>• Remove probes related with chromosome Y or X<br>• Remove technical control probes<br>• Remove probes with SNPs, CpG or SBE in target sequence<br>• Remove samples without metadata information	Logit-transformed (M values)	Non-Parametric ComBat *	311,135 x 95
**Skin Cutaneous Melanoma**							
**mRNA**	Illumina HiSeq 2000	20,531 x 428	RSME + UQ normalization	• Remove replicated samples<br>• Remove features with 0 in all samples<br>• Remove samples taken after 1 year of diagnosis<br>• Remove cases different of "Primary Tumor" or "Regional Lymph Node"	Log 2-transformed	Parametric ComBat	20,225 x 104
**miRNA**	Illumina HiSeq 2000	1,046 x 428	RPM	• Remove features with missing values<br>• Remove features with 0 in all samples<br>• Remove samples taken after 1 year of diagnosis<br>• Remove cases different of "Primary Tumor" or "Regional Lymph Node"	Log 2-transformed	Parametric ComBat	898 x 104
**Methylation**	Illumina Human Methylation 450K	485,577 x 428	Beta values	• Remove features with missing values<br>• Remove probes related with chromosome Y or X<br>• Remove technical control probes<br>• Remove probes with SNPs, CpG or SBE in target sequence<br>• Remove samples taken after 1 year of diagnosis<br>• Remove cases different of "Primary Tumor" or "Regional Lymph Node"	Logit-transformed (M values)	Non-Parametric ComBat *	305,180 x 104

Supplementary Table 2.

	Glioblastoma Multiforme		Skin Cutaneous Melanoma	
N	541	Missings	104	Missings
Gender				
Male	327		62 (59.6)	
Female	214		42 (40.4)	
Age (years)	59 [50-69]		58 [51-71]	
Age at diagnosis (years)	59 [49-68]		57 [50-71]	
Race		20		
White	476		100 (96.2)	
Black or African American	32		0 (0.0)	
Asian	13		4 (3.8)	
Ethnicity		78		2
Hispanic or Latino	12		2 (1.9)	
Not Hispanic or Latino	451		100 (96.2)	
Vital Status				
Alive	116		60 (57.7)	
Dead	425		44 (42.3)	
Days to death	372 [165-596]		468 [322-738]	61
Days to last follow-up	302 [143-513]		560 [293-1190]	34
Gene Expression Subtype†			NA	
Classical	149		NA	
Mesenchymal	164		NA	
Neural	89		NA	
Proneural	139		NA	
Histological Subtype			NA	
Glioblastoma Multiforme (GBM)	6		NA	
Treated primary GBM	20		NA	
Untreated primary (de novo) GBM	515		NA	
Prior Glioma			NA	
Yes	15		NA	
No	526		NA	
Karnofsky Score	80 [70 - 80]		NA	
Timing (205 NA's)		205	NA	
Post-Adjuvant Therapy	66		NA	
Pre-Adjuvant Therapy	123		NA	
Pre-Operative	122		NA	
Other	25		NA	
Primary site of disease	NA			
Distant Metastasis	NA		0 (0.0)	
Primary Tumor	NA		41 (39.4)	
Regional cutaneous or subcutaneous tissue	NA		0 (0.0)	
Regional lymph node	NA		63 (60.6)	
Melanoma ulceration	NA			26
Yes	NA		54 (51.9)	
No	NA		24 (23.1)	
Melanoma primary location know	NA			
Yes	NA		85 (81.7)	
No	NA		19 (18.3)	

Supplementary Table 3.

Variable	Description
Gene Expression Subtype	GBM molecular classification: Classical / Mesenchymal / Neural / Proneural / Unclassified
Gender	The collection of behaviors and attitudes that distinguish people on the basis of the societal roles expected for the two sexes. Male / Female
Race	A classification of humans characterized by certain heritable traits, common history, nationality, or geographic distribution.<br>White / black or african American / Asian
Ethnicity	A socially defined category of people based on common ancestral, cultural, biological, and social factors. Hispanic or latino / not hispanic or latino
Days to birth	Time interval from a person's date of birth to the date of initial pathologic diagnosis, represented as a calculated negative number of days.
Age at diagnostic	The age in years of the case at the initial pathological diagnosis of disease or cancer.
Vital status	The state of being living or deceased for cases that are part of the investigation. Alive / Dead
Days to death	The number of days from the date of the initial pathological diagnosis to the date of death for the case in the investigation.
Histological type	Glioblastoma Multiforme (GBM) / Treated primary GBM /Untreated primary (de novo) GBM
Prior glioma	Patient's history of prior cancer diagnosis
Tissue Source Site (TSS)	Centers who collects samples (tissue, cell or blood) and clinical metadata, which are then sent to a BCR. A TSS is identified by its TSS ID. Number of different TSS: 21
Karnofsky Score	An index designed for classifying patients 16 years of age or older by their functional impairment. A standard way of measuring the ability of cancer patients to perform ordinary tasks.
Timing	A time reference for the Karnofsky score and/or the ECOG score using the defined categories.<br>Post-Adjuvant Therapy / Pre-Adjuvant Therapy / Pre-Operative / Other
Batch number	A set of related analytes prepared for further analysis, numbered sequentially, from the same disease. Once a Case has been assigned to a batch, subsequent shipments from that case are assigned the same batch number as the original. Number of different batches: 24
Days to last follow up	The number of days from the date of the initial pathological diagnosis to the date of last follow up for the case in the investigation.

Supplementary Table 4.

Variable	Description
Years to birth	Numeric value to represent the calendar year in which an individual was born.
Vital status	The state of being living or deceased for cases that are part of the investigation. Alive / Dead
Days to death	The number of days from the date of the initial pathological diagnosis to the date of death for the case in the investigation.
Days to last follow-up	Number of days between the date used for index and the date the patient was seen or contacted at follow-up.
Days to submitted specimen dx	The age in years of the case at the initial pathological diagnosis of disease or cancer.
Primary site of disease	The anatomical site where the primary tumor is located in the organism.<br>Distant Metastasis / Primary Tumor / Regional cutaneous or subcutaneous tissue / Regional lymph node
Neoplasm disease stage	Stage of neoplasma. i or ii nos / stage 0 / stage i / stage ia / stage ib / stage ii / stage iia / stage iib / stage iic / stage iii / stage iiia / stage iiib / stage iiic / stage iv
Pathology T stage	The T category describes the original (primary) tumor. t0 / t1 / t1a / t1b / t2 / t2a / t2b / t3 / t3a / t3b / t4 / t4a / t4b / tis / tx
Pathology N stage	The N category describes whether or not the cancer has reached nearby lymph nodes. n0 / n1 / n1a / n1b / n2 / n2a / n2b / n2c / n3 / nx
Pathology M stage	The M category tells whether there are distant metastases (spread of cancer to other parts of the body). m0 / m1 / m1a / m1b / m1c
Melanoma ulceration	Presence of ulcers in melanoma. Yes / No
dcc_upload_date	Date of data upload
Breslow thickness	Thickness of primary tumor at initial diagnosis (mm).
Gender	The collection of behaviors and attitudes that distinguish people on the basis of the societal roles expected for the two sexes. Male / Female
Date of initial pathologic diagnosis	Date of initial pathologic diagnosis.
Radiations radiation regimen indication	Radiarion regime indicated. Yes / No
Race	A classification of humans characterized by certain heritable traits, common history, nationality, or geographic distribution.<br>White / black or african American / Asian
Ethnicity	A socially defined category of people based on common ancestral, cultural, biological, and social factors. Hispanic or latino / not hispanic or latino
Batch number	A set of related analytes prepared for further analysis, numbered sequentially, from the same disease. Once a Case has been assigned to a batch, subsequent shipments from that case are assigned the same batch number as the original. Number of different batches: 17

Supplementary Table 5.

	Component	r.JIVE	PCA-GCA (1%)	PCA-GCA (5%)	pESCA (1%)	pESCA (5%)
**Glioblastoma**						
**mRNA + miRNA**						
	Common	1	3	NA	7	1
	Dist mRNA	50	8	NA	6	4
	Dist miRNA	19	5	NA	14	1
**mRNA + Methylation**						
	Common	3	NA	NA	1	1
	Dist mRNA	17	NA	NA	1	1
	Dist methylation	25	NA	NA	8	2
**Skin Cutaneous Melanoma**						
**mRNA + miRNA**						
	Common	3	50	1	7	2
	Dist mRNA	20	15	1	12	2
	Dist miRNA	17	13	2	2	3
**mRNA + Methylation**						
	Common	4	65	1	4	1
	Dist mRNA	19	0	1	1	3
	Dist methylation	36	38	3	13	2

Supplementary Table 6.

	GBM		SKCM
**mRNA + miRNA**	Overlapping samples	Whole dataset	
mRNA dimension	7,814 x 515	7,814 x 523	9,491 x 104
mRNA significant	**1**	**4**	**216**
miRNA dimension	323 x 515	323 x 518	239 x 104
miRNA significant	**1**	**1**	**6**
mRNA-miRNA total pairs	24,665	24,665	20,225
NPC_Fisher significant pairs	397	466	529
New mRNA from NPC	**337**	**382**	**190**
New miRNA from NPC	**45**	**54**	**77**
**mRNA + methylation**			
mRNA dimension	9,620 x 83	9,620 x 523	9,564 x 104
mRNA significant	**2**	**7**	**216**
Methylation dimension	57,645 x 83	57,645 x 95	55,729 x 104
methylation significant	**1**	**0**	**12**
mRNA-methylation total pairs	57,645	57,645	55,729
NPC_Fisher significant pairs	323	207	1,418
New mRNA from NPC	**179**	**89**	**245**
New methylation sites from NPC	**322**	**207**	**1,406**

Supplementary Figures

STATegRa manuscript

Supplementary Figure 1.

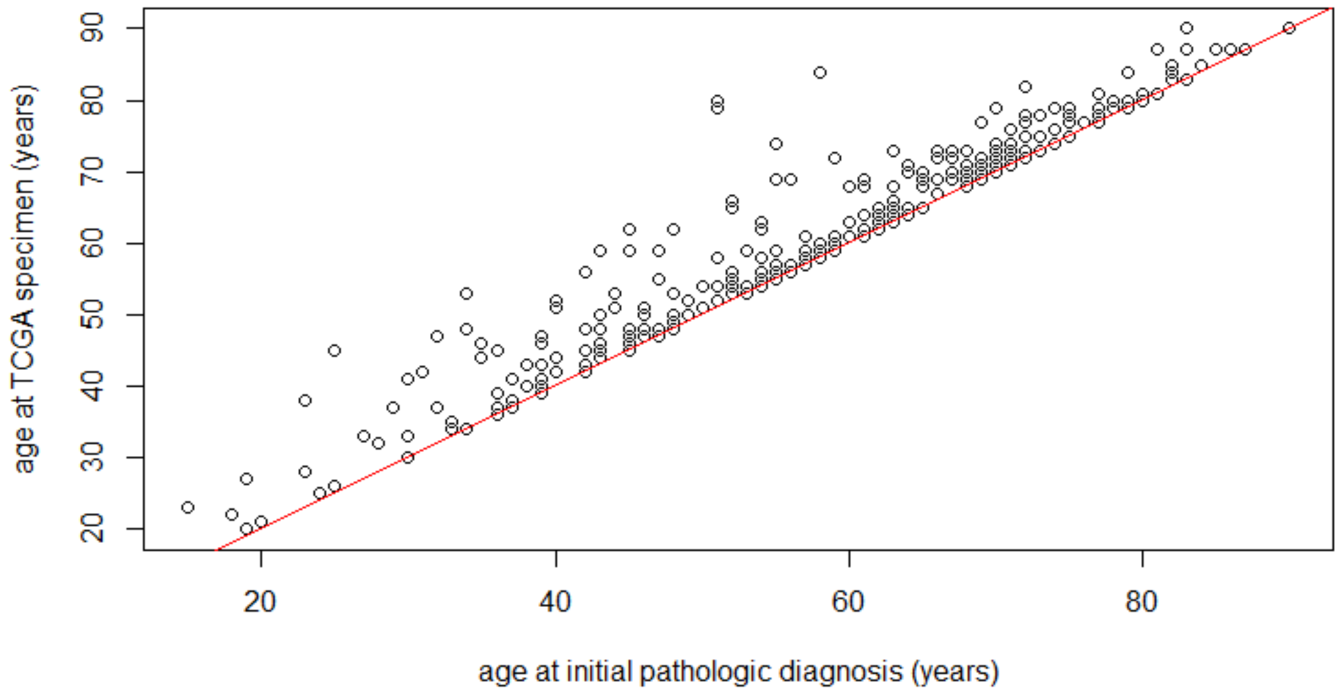

Supplementary Fig 2.

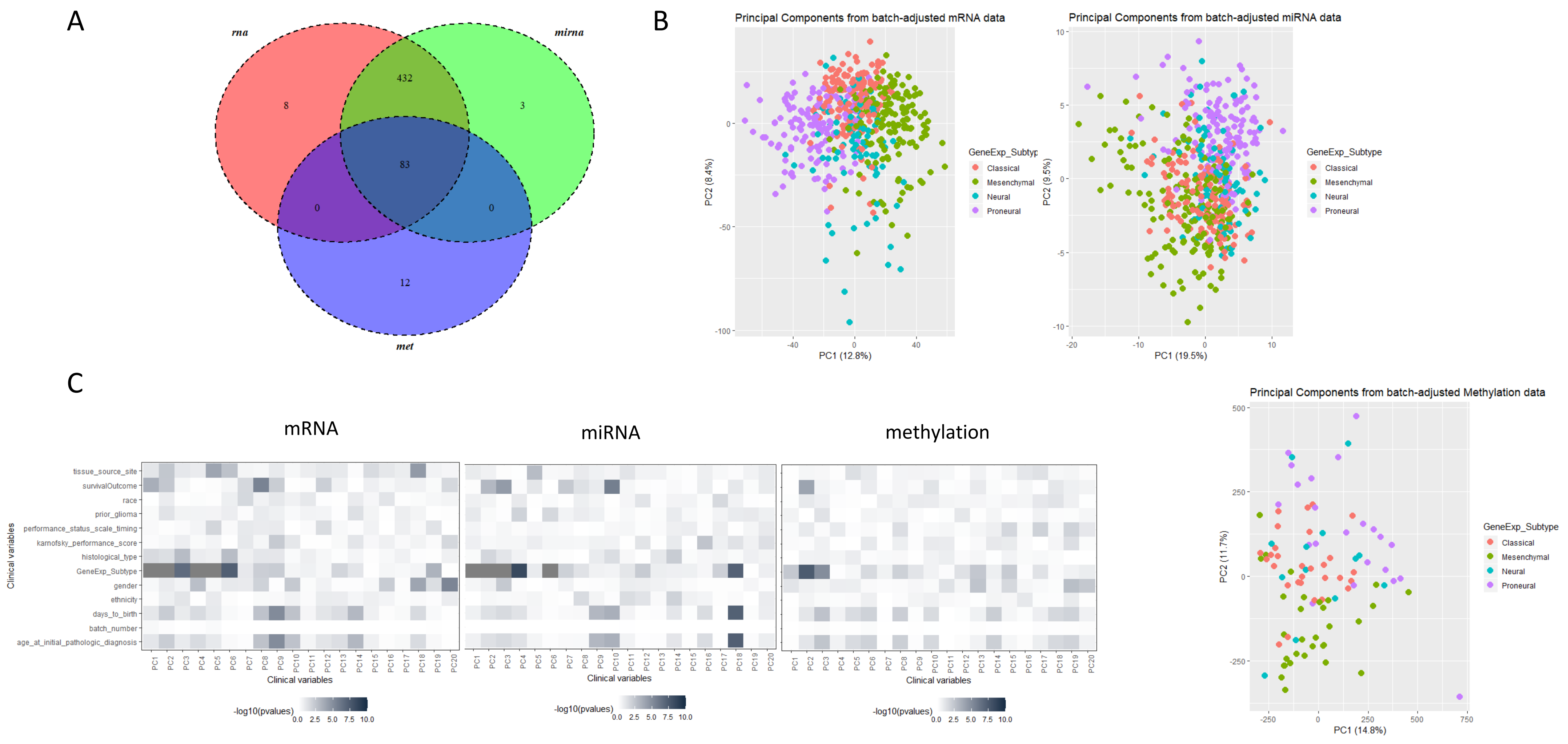

Supplementary Fig 3.

A

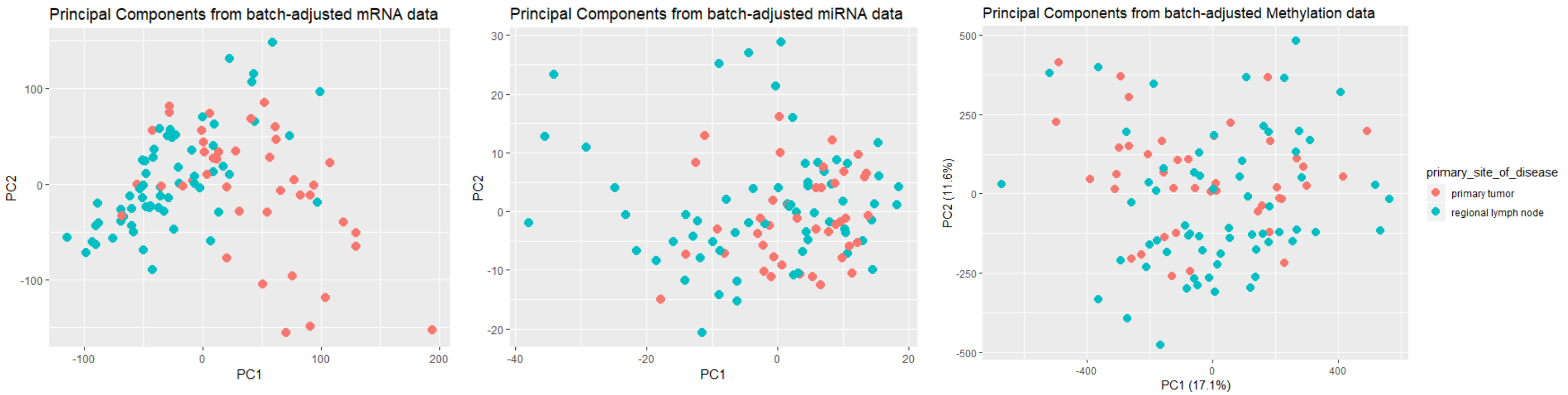

B

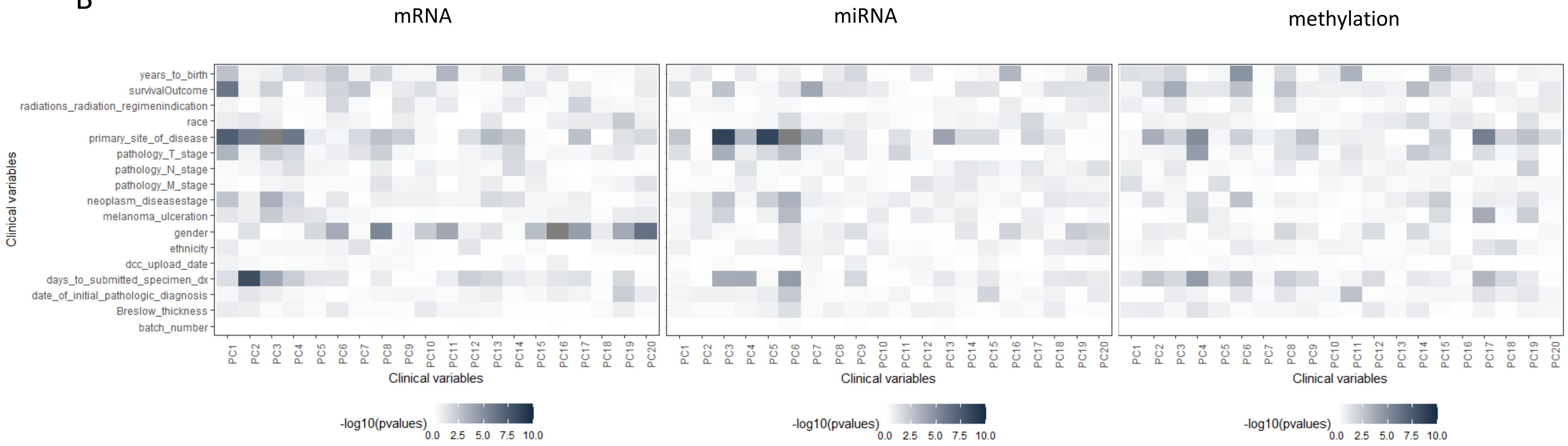

Supplementary Figure 4.

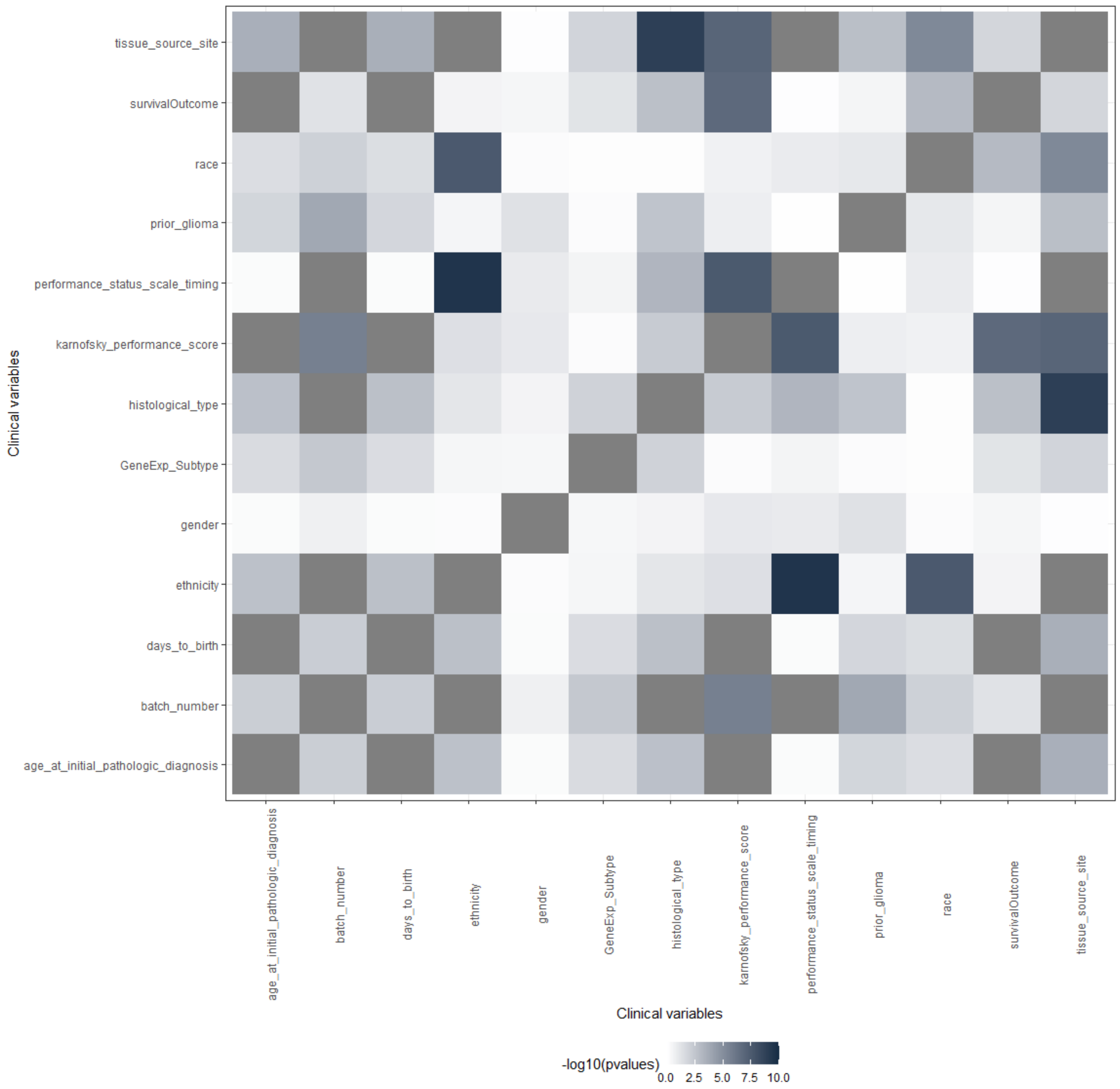

Supplementary Figure 5.

Age in GBM

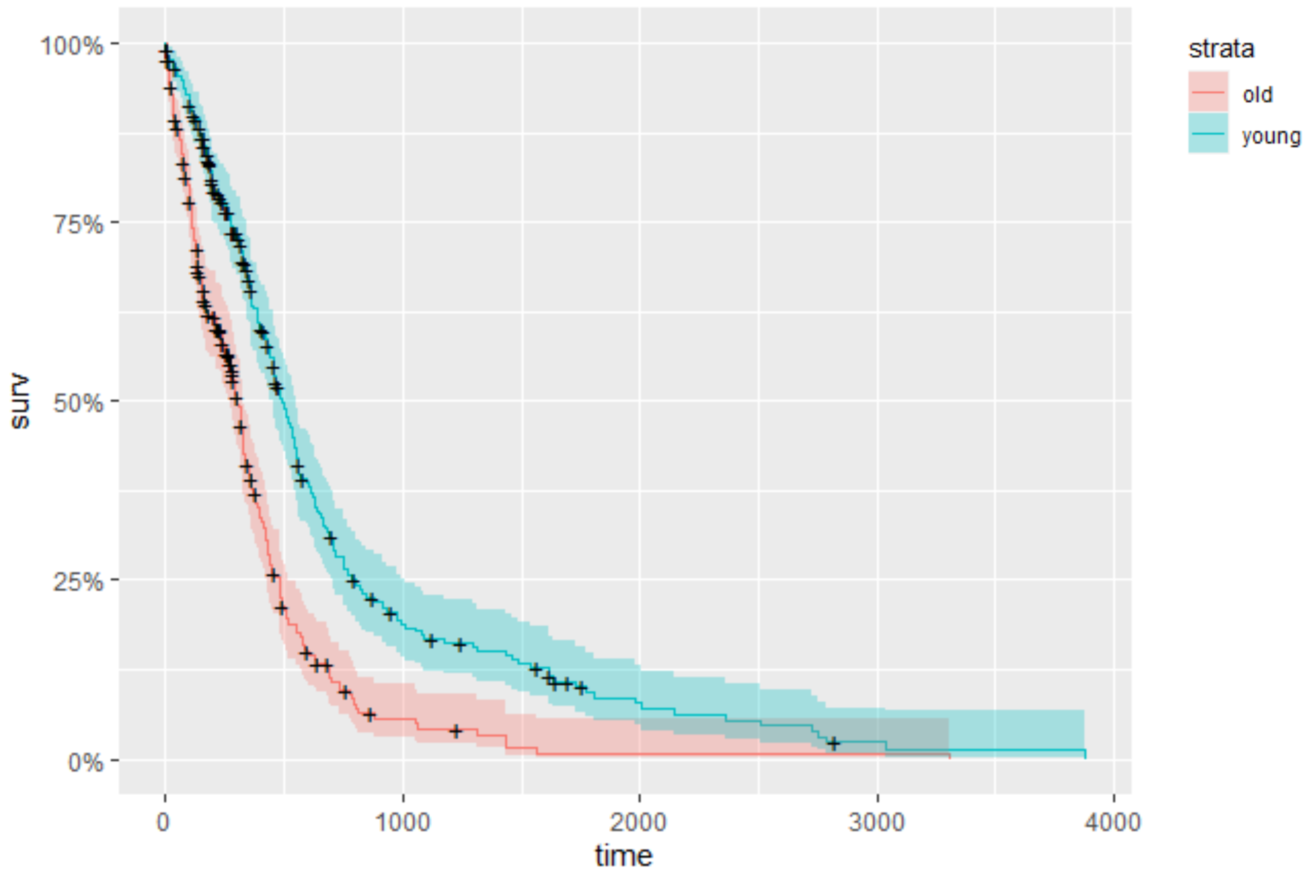

Supplementary Figure 6.

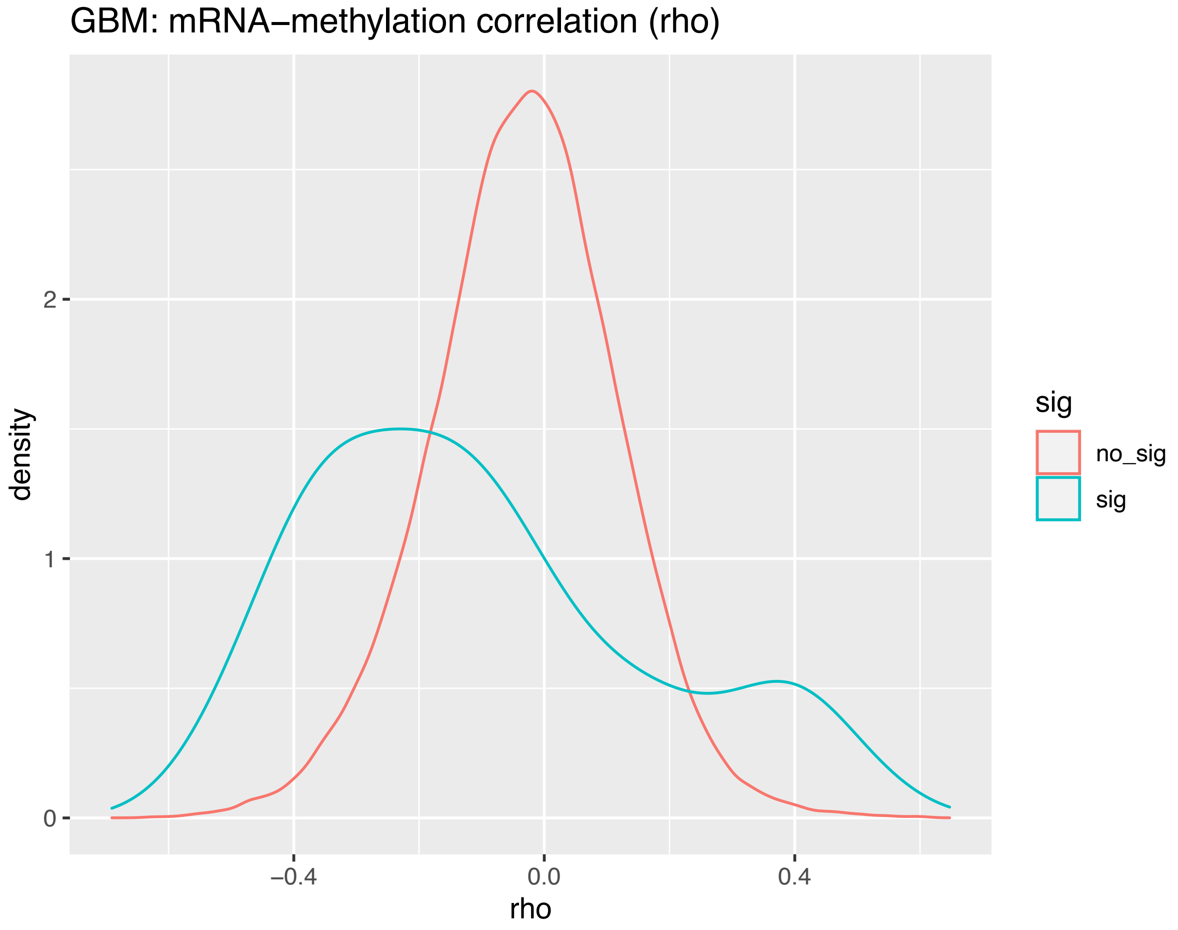

(a)

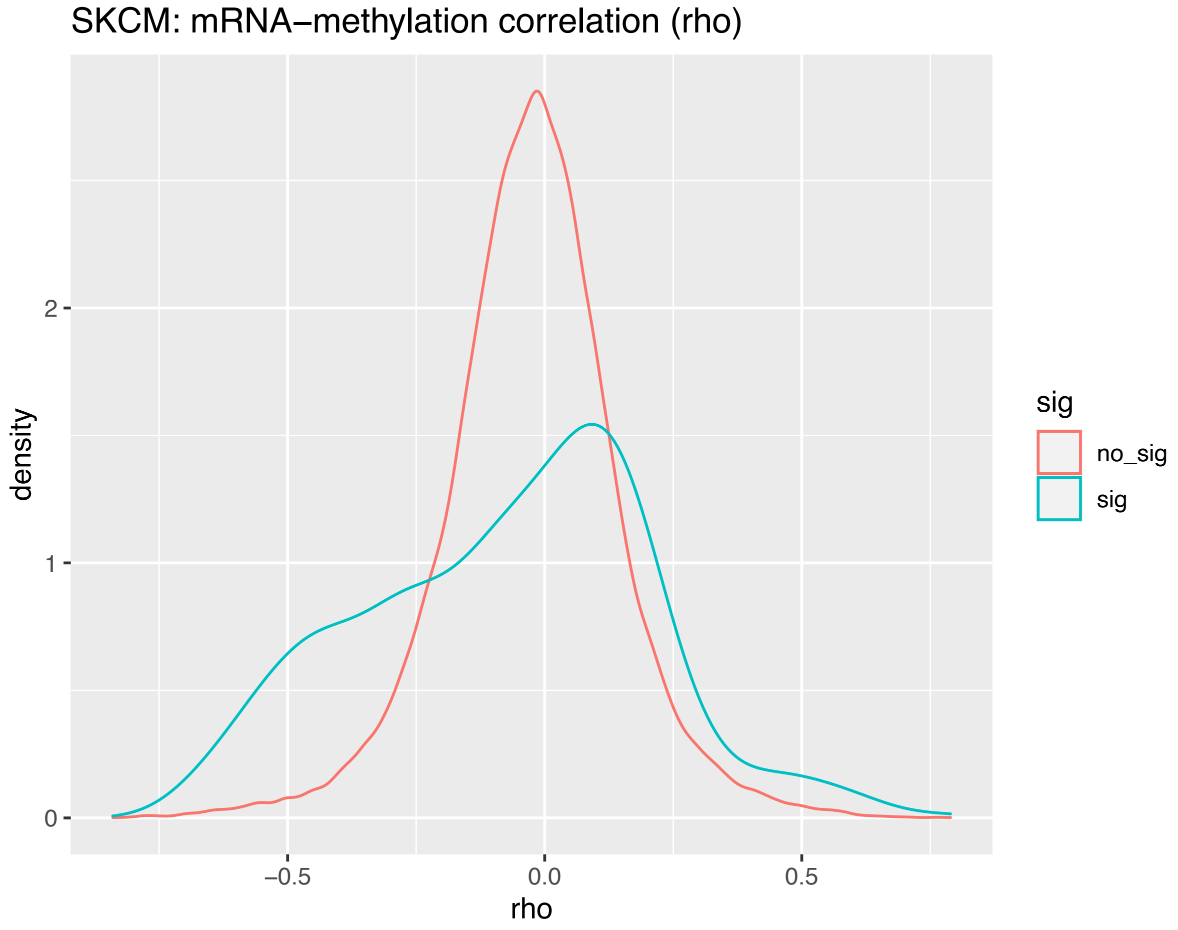

(b)
